# Recent Progress on Primary Central Nervous System Lymphoma—From Bench to Bedside

**DOI:** 10.3389/fonc.2021.689843

**Published:** 2021-08-18

**Authors:** Liang Shao, Chengshi Xu, Huijing Wu, Muhammad Jamal, Shan Pan, Sirui Li, Fei Chen, Ding Yu, Kui Liu, Yongchang Wei

**Affiliations:** ^1^Department of Hematology, Zhongnan Hospital of Wuhan University, Wuhan, China; ^2^Department of Neurosurgery, Zhongnan Hospital of Wuhan University, Wuhan, China; ^3^Department of Lymphoma Medicine, Hubei Cancer Hospital, Tongji Medical College, Huazhong University of Science and Technology, Wuhan, China; ^4^Department of Immunology, School of Basic Medical Science, Wuhan University, Wuhan, China; ^5^School of Medicine, Wuhan University of Science and Technology, Wuhan, China; ^6^Department of Radiology, Zhongnan Hospital of Wuhan University, Wuhan, China; ^7^Department of Radiation and Medical Oncology, Zhongnan Hospital of Wuhan University, Wuhan, China

**Keywords:** Primary CNS lymphoma, Bruton’s tyrosine kinase inhibitor, ASCT, CAR T, whole-brain radiotherapy

## Abstract

Primary central nervous system lymphoma (PCNSL) is a rare subtype of extra-nodal lymphoma. The high relapse rate of PCNSL remains a major challenge to the hematologists, even though patients exhibit high sensitivity to the methotrexate-based chemotherapeutic regimens. Recently, the advent of Bruton’s tyrosine kinase inhibitor (BTKi) and CAR T treatment has made more treatment options available to a proportion of patients. However, whether BTKi monotherapy should be given alone or in combination with conventional chemotherapy is still a clinical question. The status of CAR T therapy for PCNSLs also needs to be elucidated. In this review, we summarized the latest progress on the epidemiology, pathology, clinical manifestation, diagnosis, and treatment options for PCNSLs.

## Background

PCNSL is a rare type of extra-nodal B-cell non-Hodgkin lymphoma (NHL) with a focus located in the brain, leptomeninges, spinal cord, or eyes (1–3). Concerning the pathological subtype, the vast majority of PCNSLs (>95%) are diffuse large B-cell lymphomas (DLBCLs), expressing B-cell markers such as CD20, CD19, CD79a, and immunoglobulin light chains. Unlike other primary brain tumors, PCNSL is more sensitive to corticosteroids, radiotherapy, and some chemotherapeutic drugs, such as methotrexate and cytarabine (1–3). However, the most frequently used combination chemotherapy regimens for systemic DLBCL have been proven ineffective in PCNSL due to the drugs’ poor penetration to the CNS and their inability to cross the blood–brain barrier (BBB) (1, 3). Therefore, PCNSL is an aggressive disease that has a poor prognosis, with a 5-year survival rate of 30.1% (1–3).

## Epidemiology

The incidence of PCNSL was approximately 0.44/100,000 persons in the period 2009–2015. The ratio of males/females is 1–1.1 (1, 4). PCNSL develops into two different individual types: immunocompetent and immunosuppressed patients. The former is estimated to account for up to 1%–2% of NHLs and approximately 3%–5% of all primary brain tumors. The latter is found mainly in patients suffering from human immunodeficiency virus (HIV) infection, leading to the sharp increase in PCNSLs in immunosuppressed patients in recent decades (1, 5–7).

## Pathology

The vast majority of PCNSLs are DLBCLs. Approximately 10% of cases are indolent B-cell lymphomas, peripheral T-cell lymphomas, or Burkitt lymphomas (1, 2). PCNSL-DLBCL can be further subdivided into germinal center B cell (GCB) and non-germinal center B-cell (non-GCB) subtypes, depending on the gene expression profile. Primary central nervous system T-cell lymphoma (PCNSTCL) seems to benefit from high-dose methotrexate (HD-MTX), similarly to PCNSL with a DLBCL pathology (8, 9).

PCNSL-DLBCL expresses B-cell antigens such as CD79a, CD19, and CD20, as well as monotypic surface immunoglobulin light chains. However, PCNSL has a unique molecular profile that is distinct from that of systemic DLBCL. CD10 is expressed in only a minority (<10%) of cases, BCL6 protein is expressed in 60%–80%, BCL6 rearrangements are seen in ~30%, and interferon regulatory factor 4 (IRF4) is expressed in 90% of cases. BCL6 rearrangements usually predict a poor prognosis of PCNSL (10, 11). DLBCLs usually have high proliferative activity and often lack HLA molecules, probably owing to genetic loss of the HLA locus at chromosome 6p21.3. Combined whole-exome sequencing (WES) and targeted sequencing in 27 PCNSL patients identified *MYD88* mutation in 67% of patients, *CDKN2A* biallelic loss in 44%, and *CD79B* mutation in 61% (12).

An elegant study demonstrated that 86% of PCNSLs harbored oncogenic gain-of-function mutations in *MYD88* (MYD88L265P), 64% gained missense mutations in the immunoreceptor tyrosine-based activation motif domain of *CD79B*, 29% had missense mutations in the coiled-coil domain of *CARD11*, and 71% harbored missense mutations in the kinase domain of *PIM1* (13). Additionally, 29% of PCNSLs had mutations in *IRF4*, which encodes the IRF4 transcription factor, which regulates germinal center exit, class switch recombination, and plasma cell development. It is also expressed in ABC-type DLBCLs. Twenty-one percent of PCNSLs harbored mutations of *ETV6*, which was also disrupted by inactivating deletions of coding exons. Some 43% and 36% had mutations in *BTG1* and *TBL1XR1*, respectively. These genes encode transcription cofactors that regulate ETV6 activity. TBL1XR1 also modulates TLR/MYD88 signaling by increasing the clearance of NCor/SMRT transcriptional co-repressors from certain *TLR/MYD88* target genes. In these PCNSLs, all *CD79B* mutations occurred in the context of *MYD88* mutations. Similarly, the less frequent *CARD11* mutations all occurred in *MYD88* mutation-positive PCNSLs. PCNSLs with copy number loss of *TNFAIP3* had concurrent *CD79B* and *MYD88* mutations. *MYD88* mutations also occurred in association with additional potential modulators of TLR signaling such as NFKBIZ copy number gains. Furthermore, these PCNSLs often had mutations and/or exon deletions of *ETV6* and/or mutations of the transcriptional cofactors BTG1 and TBL1XR1. The majority of PCNSLs also had evidence of genomic instability as reflected by *CDKN2A* and/or *FHIT* loss and multiple CNAs. Some 7% of PCNSLs had infrequent *TP53* mutations (13).

Some novel gene mutations or unique protein expression panel findings have been reported, such as *PIM1* mutations (14), *SLIT2* variants (p. N63S, p. T590M, p. T732S) (15), p-STAT3 protein expression (16), and deregulated RelA/p65 expression (17).

## Clinical Manifestation

The most common clinical manifestation is headache, occurring in 30%–40% of patients with leptomeningeal metastases (1, 18, 19). Some patients present with personality change, cognitive decline, and weakness, which might be due to the infiltration of lymphoma cells into the white matter tracts of the corpus callosum and internal capsule. In addition, language deficits, paresis of extremities, and signs of cerebral edema have been in the patients with edematous masses. In rare cases, patients can present with gradually progressing Parkinsonism (20). In the case of infiltration of lymphoma cells into the walls of the third ventricle, the patients can present with aberrant secretion of antidiuretic hormone, insipidus, hyperphagia, hyposexuality, and psychotic thought changes. Invasion into the walls of the fourth ventricle and the brainstem by lymphoma cells, in rare cases, can lead to dysconjugate gaze, vertigo, intractable vomiting, and ataxia.

Small-vessel involvement makes patients present with fatigue and weight loss and nocturnal perspiration, hepatosplenomegaly, or unexplained pancytopenia, while multivessel invasion usually triggers “lacunar stroke” syndromes, presenting with subcortical dementia, myelopathies, and lymphocyte infiltration in the spinal fluid. When lymphoma cells infiltrate the cranial or peripheral nerve roots, patients can present with migratory pain syndromes. Fifteen percent of PCNSL is correlated with concurrent or subsequent eye involvement. Intraocular lymphoma (IOL) usually presents with ocular “floaters” and blurred vision progression in both eyes. For primary IOL patients, the risk of brain involvement is as high as 50% (1, 18, 19). PCNSL patients have shown an increased incidences of venous thromboembolism (VTE) and major bleeding (21).

## Clinical Diagnosis

### Imaging

PCNSLs are usually displayed on conventional CT scanning as lesions with hyperintensity or isointensity, and enhanced CT scanning shows obvious enhancement (22–24). However, CT scanning cannot well differentiate PCNSLs from other intracranial space-occupying diseases. To date, MRI is still the main imaging technique for the non-invasive diagnosis of PCNSLs and the imaging features of PCNSLs are mainly dependent on their histopathology. PCNSL tissues usually exhibit isointensity or hypointensity on T1WI, and isointensity or relative hyperintensity or hypointensity on T2WI. The appearance of some unique features, such as “incision”, “fist”, or “angular” signs, on enhanced MRI are helpful for the diagnosis of PCNSL. Additionally, linear enhancement along the periventricular area is indicative of PCNSL. Diffusion-weighted imaging (DWI) is a widely used non-contrast MRI sequence for the assessment of PCNSLs (22, 25). Apparent diffusion coefficient (ADC) values are clinically useful for the differentiation of PCNSLs from other primary brain tumors because the ADC values of PCNSLs are significantly lower than those of glioblastomas. Dynamic susceptibility contrast perfusion MR imaging (DSC-MRI) measures relative cerebral blood volume (rCBV), which has potential utility for PCNSL diagnosis and clinical outcomes. Assessment of rCBV is effective in the non-invasive differentiation of PCNSLs from glioblastomas. In detail, rCBV values in PCNSLs are lower than those observed within glioblastomas (22, 25). Dynamic contrast-enhanced (DCE) perfusion MR imaging, with the ability to capture unique features of the vascular microenvironment, enables it to potentially differentiate lymphomas from other brain tumors. Patients with lymphoma have shown higher values of Ktrans, a parameter of DCE-MRI, than glioblastoma and metastases.

### Histopathology and Immunohistochemistry

More than 98% of PCNSLs are malignant NHLs of the B-cell type (1). The tumor contains perivascular B cells expressing pan-B-cell markers such as CD19, CD20, and CD79a (1–4). Infiltrated T-cell CD3 expression is always present in infiltrated T cells. In non-immunosuppressed patients, the majority of the tumors are categorized as DLBCL. In immunosuppressed individuals, DLBCL and Burkitt-like or atypical tumors are the most common tumors types that occurred. Other histological subtypes, all rare cases, include lymphoplasmacytic lymphoma, follicular lymphoma, marginal zone lymphoma of the dura, and primary T-cell lymphoma of the brain. The histological diagnosis determines the standard of care. The existence of clonal proliferation in brain tissues can be confirmed by immunoglobulin gene rearrangement analysis or immunohistochemistry (IHC). Dense and rapidly growing tumors usually express high levels of Ki67 (> 50%) (26, 27).

## Treatment

### Methotrexate-Based Chemotherapy

Single-drug and multi-drug methotrexate-based regimens have been widely used in the treatment of PCNSL. The methotrexate dose ranges from 1 to 8 g/m^2^ with 78% of patients receiving at least 3 g/m^2^/injection (1–3). Interestingly, HD-MTX has also been administered to the vast majority of the oldest patients aged over 80 years (84%), while 39% of them frequently received the drug at a reduced dose (≥3 g/m^2^/injection). However, the incremental benefit of additional chemotherapeutic drugs is unclear and the selection of the specific drugs to incorporate into the multi-drug regimens has been mostly empirical. Notably, intravitreal MTX seems to be a safe and effective treatment for relapsed PIOL after systemic chemotherapy and radiotherapy (28). Generally, PCNSL has a cure rate below 40% with MTX-based regimens and is subject to late recurrences (1, 29–32). In their analysis of 31 studies published between 1992 and 2019, Yu et al. have reported that PCNSL patients achieved a pooled CRR of 41% across all HD-MTX-based regimens (33). Additionally, three- and four-drug regimens achieved better CRRs than HD-MTX monotherapy (33).

### Immunotherapy

#### BTK Inhibitor

A preclinical study on ibrutinib brain distribution showed that the maximal concentrations of the BTK inhibitor ibrutinib in plasma and brain were close, suggesting that ibrutinib rapidly crosses the BBB in 0.29 h (0.2–0.32 h) [median (min–max)] (34). Ibrutinib brain exposure was also associated with the drug dosage and was correlated with plasma exposure. The average AUC0−t brain to AUC0−t plasma ratio average of ibrutinib reached 0.7, and ibrutinib accumulated in the ventricle area.

By using orthotopic xenograft models that were established by injecting lymphoma cells into the brain parenchyma of athymic mice, Jiménez et al. demonstrated that selinexor in combination with ibrutinib could effectively suppress tumor growth and prolong the survival of CNS lymphoma mice (35). Tumor cells in their brains were accompanied by tumor-associated macrophages (TAMs) and T cells. These M2-like TAMs preferentially expressed PD-1 and SIRPα. Interestingly, the combination treatment with selinexor and ibrutinib benefited an anti-tumoral immune response by shifting the polarization towards pro-inflammatory M1-like macrophages and reducing PD-1 and SIRPα expression in the remaining M2-like TAMs.

A phase I clinical trial (NCT02315326) with ibrutinib for R/R CNS lymphoma patients was performed. Clinical responses to ibrutinib were observed in 10/13 (77%) PCNSL patients, including 5 with complete remission (CR) (36). Ibrutinib was well-tolerated, with manageable adverse events, including hyperglycemia, thrombocytopenia, anemia, and hypertriglyceridemia. No dose-limiting toxicity (DLT) was observed in the cohort during the dose-escalation phase of the study. Four patients developed grade 4 toxicities, including neutropenia, sepsis, and lymphopenia. Treatment was discontinued in one patient due to a fungal infection. The only PCNSL patient with complete ibrutinib resistance exhibited a mutation within the coiled-coil domain of caspase recruitment domain family member 11 (*CARD11*), a known ibrutinib resistance mechanism. Incomplete tumor responses were correlated with mutations in *CD79B*. *CD79B*-mutant PCNSLs exhibited enrichment of mammalian target of rapamycin (mTOR)-related gene sets and increased staining for phosphatidylinositol 3-kinase (PI3K)/mTOR activation markers. Inhibition of the PI3K isoforms p110α/p110δ or mTOR in combination with ibrutinib obviously triggered cell death in *CD79B*-mutant PCNSL cells.

In another clinical study (NCT02203526), ibrutinib was given in a 14-day window (from day −14 to day −1) before the administration of other chemotherapeutic drugs to test its single-agent activity against the disease in 18 patients (37). Out of these patients, 17 showed disease reductions, and 83% achieved a PR. Two refractory patients normalized their research FDG-PET scans. Among 9 patients with CSF involvement, 22% became negative by flow cytometry. The response rate to ibrutinib was 91% in patients who were on pre-treatment steroids. Of the 18 ibrutinib-window patients, 2 patients experienced grade 5 pulmonary/CNS aspergillosis infection, and 1 patient developed grade 3 hyponatremia. This study provides the evidence of the efficacy of ibrutinib monotherapy against PCNSL.

Clinical data from phase I/II clinical trials by the Australasian Lymphoma Alliance/MD Anderson Cancer Center have shown that the objective response rate (ORR) was 58% (CR 55%) in 33 enrolled relapsed/refractory CNSL patients (9 PCNSL and 24 SCNSL) treated with ibrutinib (38). The median progression-free survival (PFS) and overall survival (OS) among PCNSL patients were both 3.1 months, while the median PFS and OS of SCNSL patients were 10.2 and 11.5 months, respectively. The most common toxicities were atrial fibrillation (in 2 patients) and cytopenia (in 1 patient).

#### PD-1/PD-L1/2

In recent years, immune checkpoint inhibitors such as PD-1 and PD-L1/L2 inhibitors have been shown to be potential treatment options in lymphoma (39). A total of 35.7% of patients (35/98) were PD-L1^+^ on tumor cells (tPD-L1^+^), and 48% were PD-L1^+^ on tumor and non-tumor cells (tmPD-L1^+^). The number of tumor-infiltrating lymphocytes (TILs) was greater in tmPD-L1^+^ patients than in tmPD-L1^−^ patients. tPD-L1^+^ and tmPD-L1^+^ patients tended to have poor performance status. In contrast, the numbers of CD8^+^ and PD-1^+^-TILs tended to be higher in patients with good performance status and MYC/BCL2 negativity. Patients with tPD-L1^+^ exhibited a worse OS, and those with increased CD8^+^ or PD-1^+^ TILs achieved a better OS. Tumor PD-L1 expression and the number of PD-1^+^TILs were independent prognostic factors. tPD-L1^+^ patients who presented with a small number of CD8^+^ or PD-1^+^TILs exhibited a worse prognosis, and tPD-L1^−^patients with a large number of CD8^+^ or PD-1^+^TILs had the best prognosis. In the validation group, increased CD8^+^ or PD-1^+^TILs were significantly associated with prolonged survival, but PD-L1 had no prognostic significance. In conclusion, PD-L1 is frequently expressed in tumor cells and the immune microenvironment of PCNS-DLBCL and is correlated with increased TILs. Therefore, PD-L1, CD8^+^ TILs, and PD-1^+^TILs have potential as prognostic biomarkers and therapeutic targets in PCNS-DLBCL.

Similarly, Ou et al. reported that high PD-L1 expression was seen in 37.5% of PCNSL patients, and intermediate expression was observed in 29.2%. Some 33.3% lacked PD-L1 expression (14). PD-1 expression was observed in 12/14 tumors (85.7%) and was uncorrelated with PD-L1 expression. A tumor mutational burden (TMB) of greater than or equal to 5 mutations per megabase occurred in 41/42 tumors, with 19% exhibiting high TMB, 71.4% exhibiting intermediate TMB, and 9.5% exhibiting low TMB. No samples had MSI. Twenty-six genes showed mutations, most frequently *MYD88* (81%), *CD79B* (55%), and *PIM1* (55%). Among the 7 patients tested by RNA sequencing, one *ETV6-IGH* fusion was found. Overall, 18/48 samples expressed high PD-L1, and 38/42 samples showed intermediate to high TMB.

Cho et al. measured soluble PD-L1 (sPD-L1) in the serum of 68 patients with newly diagnosed DLBCL-PCNSL (40). The median level of serum sPD-L1 in the PCNSL group was higher than that in the healthy control group. Disease relapse was more frequent in the high-sPD-L1 patients. The OS and PFS for the high-sPD-L1 cohorts were significantly lower than those in the low-sPD-L1 cohort. PD-L1-positive tumor cells were found in 35 patients, and the extent of PD-L1-postive tumor cells was positively associated with the serum sPD-L1 level. Correlation analysis showed that the serum level of IL-7 was correlated with the serum level of sPD-L1.

In 17 EBV^+^ cases, PD-L1 was expressed in both lymphoma cells and TAMs in 12 patients, but in only TAMs in 4 cases (41). Out of 22 EBV^−^ patients, PD-L1 was detected in both lymphoma cells and TAMs in 11 patients, but only TAMs in 4 patients. There was no significant difference in the number of FOXP_3_^+^ lymphocytes between EBV^+^ and EBV^−^ patients. However, there were significantly higher numbers of PD-1^+^ lymphocytes in the former and significantly higher numbers of TIA-1^+^ lymphocytes in the latter. The combined data indicate that the expression of PD-L1 by lymphoma cells and TAMs mediates the trafficking of TILs, which may explain the immune escape process of PCNSLs. In addition, EBV infection appears to affect the trafficking mechanism of TILs and thus may play an important role in the microenvironment immunity of these tumors.

A seemingly contradictory dataset on 70 PCNSL patients showed that the lymphoma cells expressed PD-L1^low/−^ and PD-L2^low/−^, but macrophages expressed PD-L1 and PD-L2 in the majority of the patients (42). The median percentage of PD-L2-positive cells was significantly higher among peritumoral macrophages than among intratumoral macrophages. PD-L1 expression on macrophages was significantly associated with biological factors and longer OS. It predicts a favorable prognosis when expressed on peritumoral macrophages.

Next-generation sequencing (NGS) for transcript variant detection and multivariable analyses on 84 transcript variants relating to Th-1/Th-2 balance and stimulatory and inhibitory checkpoints in 31 PCNSLs has shown that Th-1^low^, Th-2^high^, and stimulatory checkpoint^high^ predicted a poor prognosis (43). Furthermore, Th-1^high^Th-2^low^ was correlated with a good prognosis. CD40-001^high^ and CD70-001^high^ are stimulatory gene expression patterns and LAG3-001^high^, PDCD1 (PD-1)-001/002/003^high^, and PDCD1LG2 (PD-L2)-201^low^ were inhibitory gene expression patterns that predicted a poor prognosis. Th-1^high^Th-2^low^ and Th-1^low^Th-2^high^ were correlated with stimulatory checkpoint^low^ as CD70-001^low^ and inhibitory checkpoint^low^ as HAVCR2 (TIM-3)-001^low^ and PDCD1LG2-001/201^low^, respectively. Specific variants of CD274 (PD-L1)-001 and PDCD1-002 showed severe hazard ratios. In particular, PDCD1-002^high^ predicted a poor prognosis, as did PDCD1-001/003^high^, PDCD1LG2-201^low^, and LAG3-001^high^. These results suggest that the expression of transcript variants of PDCD1 and PDCD1LG2 affect the Th-1/Th-2 balance and might be predictive of the prognosis in PCNSL.

A preclinical study determined the therapeutic effects of anti-PDCD1 (anti-PD-1) on CNS lymphoma in a murine model. Anti-PDCD1 treatment significantly decreased tumor growth, resulting in prolongation of survival (44). In detail, no evidence of tumor existence was shown in half of the mice treated with anti-PDCD1 that went into CR and IHC, indicating a potential cure. Mechanistically, IHC showed that administration of anti-PDCD1 treatment resulted in a significant increase in CD8^+^ lymphocyte infiltration in the tumor as well as non-tumor-bearing areas of the brain.

In a clinical study, nivolumab (PD-1/L1 monoclonal antibody) displayed activity in five R/R PCNSL and primary testicular lymphoma (PTL) patients (45). Four patients had disease relapse and one had primary refractory disease following standard chemotherapy. All five patients had clinical and radiographic responses to off-label treatment with nivolumab, and three patients remained progression free at 13 to 17 months. Another open-label phase II clinical trial testing the maintenance therapy of nivolumab for newly diagnosed PCNSL with persistent circulating tumor DNA in the CSF after completion of MTX-based first-line induction chemotherapy is ongoing (NCT04401774) (**Table 1**). Other ongoing clinical trials (NCT04899427 and NCT04831658) will provide more information (**Table 1**).

**Table 1:** Registered ongoing clinical trials for the treatment of PCNSL on ClinicaTrial.gov.

Clinical trial number and study start time	Agents tested	Estimated enrollment	Trail setting	Primary endpoint	Ref.
NCT02315326	Ibrutinib/	109	A non-randomized phase I/II study	1) The maximum tolerated dose (MTD) of ibrutinib (phase I)	Ref. (36)
Year: 2014	Ibrutinib	R/R PCNSL		2) PFS (phase II)	
	combined with HD-MTX	or SCNSL		3) MTD of ibrutinib in combination with HD-MTX	
NCT02203526	1) Ibrutinib	68	A phase I study	1) MTD of ibrutinib when given with TEDD-R	Ref. (37)
Year: 2014	2) TEDDI-R	PCNSL		2) Safety and feasibility in untreated PCNSL patients	
				3) CR rate in untreated PCNSL patients	
				4) MTD of ibrutinib with anti-fungal prophylaxis when given with TEDD-R	
NCT04899427	Orelabrutinib combined with PD-1 inhibitor	32	A prospective multicenter phase II study	ORR	/
Year: 2021		R/R PCNSL			
NCT04831658	Orelabrutinib combined with PD-1 and fotemustine	40	A prospective clinical study	CR rate	/
Year: 2021		PCNSL			
NCT04401774	PD-1/L1 inhibitor	25	An open-label phase II trial	1) Frequencies of toxicities	/
Year: 2020	(Nivolumab)	PCNSL		2) cfDNA conversion rate in CSF	
NCT04609046	Lenalidomide combined with MTX, Rituximab, and Nivolumab (Nivo-MR2)	27	A phase I trial	1) Maximum tolerated dose (MTD)	/
Year: 2021		PCNSL		2) Proportion of evaluable patients who are able to stay on maintenance therapy	
NCT04443829	CD19 CAR T cells	12	A single-center non-randomized, open-label phase I clinical trial	1) Toxicity evaluated by the incidence of grade 3–5 toxicity causally related to the ATIMP	/
Year: 2021		R/R PCNSL		2) Feasibility of manufacturing CD19 CAR T cells evaluated by the number of therapeutic products generated	
NCT04608487	CD19 CAR T cells with Axicabtagene Ciloleucel (Axi-cel)	18	A phase I clinical trial	Number of participants with treatment-related adverse events as assessed by CTCAE version 5.0	/
Year: 2020		R/R PCNSL or SCNSL			
NCT04134117	Tisagenlecleucel (CD19-targeted CAR T Cells)	6	A pilot study	Number of participants with treatment-related adverse events as assessed by CTCAE criteria and ASTCT 2018 (CRS/NT)	/
Year: 2019		PCNSL			
NCT03484702	JCAR017	116	A single-arm, multi-cohort, multicenter, phase II study	1) ORR of JCAR017 in subjects with non-Hodgkin lymphoma (NHL; including secondary central nervous system involvement)	/
Year: 2018		aggressive B-cell non-Hodgkin lymphoma		2) ORR of JCAR017 in R/R PCNSL	
				3) AEs in subjects intended to be treated as outpatients	

These data suggest a potential role for anti-PD1 or PD-L1 monoclonal antibodies in the treatment of PCNSLs. However, more clinical evidence is needed to further confirm the efficacy of immune checkpoint inhibitors in PCNSLs.

#### Lenalidomide

Lenalidomide is a second-generation immunomodulatory agent suppressing the growth and survival of lymphoma cells *via* multiple mechanisms, including alteration of the lymphoma cell microenvironment and stimulation of T and NK cell expansion (46). Lenalidomide enhances the antibody-dependent cell-mediated cytotoxicity of rituximab and may overcome rituximab resistance in NHL (46, 47). Lenalidomide also has cell-autonomous cytotoxic effects on lymphoid tumors, including antagonism of IRF4 and MYC pro-survival signals (46–48). A study provided evidence that for elderly patients with relapsed PCNSL, lenalidomide induced a confirmed CR in two of six patients (49).

In a phase I trial (NCT01542918) of lenalidomide/rituximab plus outcomes of lenalidomide maintenance in R/R CNS lymphoma, 14 patients with refractory CD20^+^CNS lymphoma were enrolled (50). The safety, efficacy, and cerebrospinal fluid (CSF) penetration of lenalidomide at 10-, 15-, and 20-mg dose levels were determined. Nine patients achieved a response better than PR with lenalidomide monotherapy, maintaining the response for 9 months, and four maintained a response for 18 months. Median PFS for lenalidomide/rituximab was 6 months. The CSF/plasma partition coefficient of lenalidomide was 20% at the 15- and 20-mg doses. Changes in CSF interleukin-10 at 1 month correlated with the clinical response and the response duration to lenalidomide. Metabolomic profiling of CSF identified novel biomarkers, including lactate, and implicated indoleamine-2,3 dioxygenase activity with CNS lymphoma progression while taking lenalidomide. These data suggest that lenalidomide could penetrate the ventricular CSF and is active in monotherapy against relapsed CNS lymphomas.

A prospective phase II study (NCT01956695) enrolled 45 patients with R/R DLBCL-PCNSL or primary vitreoretinal lymphoma (PVRL) (51). The induction therapy consisted of eight 28-day cycles of R2 (rituximab 375 mg/m^2^, iv, d1; lenalidomide 20 mg/day, d1–21 for cycle 1; and 25 mg/day, d1–21 for cycles 2–8). In responding patients, the induction therapy was followed by a maintenance treatment comprising twelve 28-day cycles of lenalidomide alone (10 mg/day, d1–21). The primary endpoint was the ORR at the end of induction. Of the 45 enrolled PCNSL patients, the ORR at the end of induction was 35.6% and 32.0% in the intent-to-treat analysis, including 13 CR/uCR and 3 PR. The best responses were 18 CR/uCR (40%) and 12 PR (27%) during the induction phase. The maintenance treatment was started in 18 patients, and 5 patients completed it. With a median follow-up of 19.2 months, the median PFS and OS were 7.8 months and 17.7 months, respectively. No unexpected toxicity was observed. The R2 regimen showed significant activity in R/R PCNSL and PVRL patients. These results support assessments of the efficacy of R2 combined with methotrexate-based chemotherapy as a first-line treatment for PCNSL.

The combination of PD-1 monoclonal antibody (nivolumab) with lenalidomide might be a potential treatment choice for R/R PCNSL (52, 53). An ongoing phase I clinical trial (NCT04609046) is evaluating the best dose, clinical benefits, and/or side effects of lenalidomide when combined with nivolumab and common drugs (rituximab and MTX) in these diseases.

Taken together, the evidence suggests that lenalidomide alone or combined with anti-CD20 antibody/PD-1 monoclonal antibody shows clinical efficacy for R/R PCNSL.

#### Temozolomide

A phase II study (NCT01458730) by the Nordic Lymphoma Group tested de-escalating induction and introducing temozolomide (TMZ) maintenance in the elderly PCNSL patients (54). The ORR was 69.9% in the younger and 80.8% in the elderly cohorts, respectively. With a median follow-up time of 22 months, the 2-year OS probability was 60.7% in patients ≤ 65 years and 55.6% in those aged > 65 years. The estimated 2-year PFS was 33.1% in patients aged < 65 years and 44.4% in the elderly group, respectively. The median duration of response was 10 months in the younger cohort and was not reached in the elderly cohort. Unfortunately, 4 patients aged 64–75 years died from treatment-related complications. Survival in the two cohorts was similar despite a de-escalation of induction treatment in patients > 65 years. The elderly patients receiving maintenance TMZ gained a longer duration of response than the younger cohort.

In a study of induction chemotherapy with methotrexate, rituximab, and TMZ, followed by whole-brain radiotherapy and post-irradiation TMZ treatment for PCNSL (NRG Oncology RTOG 0227), 13 and 53 patients were enrolled in phase I and II studies, respectively. The maximum tolerated dosage of TMZ was 100 mg/m^2^. The main dose-limiting toxicities were hepatic and renal (55).

In a prospective, multicenter phase I study, Chiesa et al. tested the maximum tolerated dose (MTD) of TMZ concurrent with radiotherapy (RT) after HD-MTX for 9 newly diagnosed PCNSL (56). In detail, eligible patients were treated with induction therapy of HD-MTX (3.5 g/m^2^), followed by consolidation with concomitant RT and escalating TMZ (50–60–75 mg/m^2^/day, days 1–5/cycle). Six patients received two cycles of HD-MTX, while three received only one cycle because of hepatic or renal toxicity. All nine patients completed chemoradiotherapy without interruptions. No DLT events were reported. TMZ appears to be tolerable at a dose of 75 mg/m^2^/day when administered concomitantly with radiotherapy after HD-MTX.

In a retrospective study, Chen et al. compared the efficacy and toxicity of HD-MTX plus TMZ (MT regimen) with rituximab plus MT (RMT regimen) in 62 untreated PCNSL patients (57). Thirty-two patients were given RMT as induction therapy, and 30 received the MT regimen. The ORRs were 93.7% and 69.0% in the RMT and MT groups, respectively, while CRs were 53.2% and 27.6% in these two groups. The 2- and 5-year PFS rates in the RMT group were 81.3% and 53.3%, respectively. The 2- and 5-year PFS rates in the MT group were 46.5% and 29.1%, respectively. Notably, the 2- and 5-year OS rates were 82.3% and 82.3%, respectively, for the RMT group and 65.7% and 50.0%, respectively, for the MT group. These data suggest that the RMT regimen may be a feasible and safe therapeutic choice for the front-line treatment of PCNSL.

Another study detected the efficacy and tolerability of TMZ maintenance followed by an R-MPV regimen (rituximab, methotrexate, procarbazine, and vincristine) for PCNSL in 10 patients (58). In detail, TMZ was given as a single agent at the maintenance dose of 150 mg/m^2^ daily on days 1–5 in cycle 1 and increased to 200 mg/m^2^ for subsequent cycles 2–6, unless limited by toxicity, with the intention of giving at least six cycles. The median PFS was 57 months, and the 2- and 5-year PFS rates were 67% and 33%, respectively. The median OS was 63 months, and the 2- and 5-year OS rates were 88% and 57%, respectively. TMZ was well tolerated, with the most common toxicity of grade ≥3 being thrombocytopenia, in three patients.

An open-label, randomized phase II trial (NCT00503594), done in 13 French institutions, enrolled immunocompetent patients who had neuroimaging and histologically confirmed newly diagnosed primary CNS lymphoma and were aged 60 years or older (59). Patients were assigned (1:1) to receive a TM regimen (MTX 3.5 g/m^2^; TMZ 150 mg/m^2^) or an MPVC regimen (MTX 3.5 g/m^2^; procarbazine 100 mg/m^2^; vincristine 1.4 mg/m^2^; cytarabine 3 mg/m^2^). Of all ninety-five patients, 48 were randomly assigned to the TM group and 47 to the MPVC group. The 1-year PFS was 36% in the MPVC group and 36% in the TM group; the median PFS was 9.5 and 6.1 months, respectively. The ORRs were 82% and 71% in MPVC and TM groups, respectively. The median OS was 31 and 14 months in the MPVC and TM groups. No differences were observed in toxic effects between the two groups. The most common grades 3 and 4 toxicities in both groups were liver dysfunction (4% in the TM group *vs*. 38% in the MPVC group), lymphopenia (29% *vs*. 30%), and infection (13% *vs*. 15%). A total of 69% and 55% of patients died in the MT and MPVC groups, respectively. Quality-of-life evaluation (QLQ-C30 and BN20) showed improvements in most domains compared with baseline in both groups. Prospective neuropsychological testing showed no evidence of late neurotoxicity. The efficacy endpoints tended to favor the MPVC group. Both regimens were associated with similar, moderate toxicity, but the quality of life improved with time, suggesting that pursuing treatment in these poor prognosis patients is worthwhile.

A prospective multicenter phase II trial (NCT00248534) using rituximab and TMZ in immunocompetent patients with progressive or recurrent PCNSL was done (60). The treatment protocol contained an induction therapy with rituximab (750 mg/m^2^) on days 1, 8, 15, and 22 and TMZ (150 mg/m^2^) on days 1–7 and 15–21, followed by six cycles of consolidation therapy with TMZ (150–200 mg/m^2^ ×5/28 days), followed by maintenance therapy with methylprednisolone (1 g, iv, every 28 days) until disease progression. Sixteen patients were enrolled, and a CR was seen in 2/14 (14%) evaluable patients. The median follow-up time was 37 months, and the median PFS was 7 weeks. The median OS was not reached. Treatment was well-tolerated.

Thirty-one patients received induction therapy with the MT-R regimen (MTX 8 g/m^2^, day 1, ×8 cycles; rituximab 375 mg/m^2^, day 3, cycles 1–6; TMZ 150 mg/m^2^, days 7–11, odd cycles), followed by consolidation therapy with the EA regimen (etoposide 5 mg/kg, iv, days 1–4 ×8 doses; cytarabine 2 g/m^2^, iv, days 1–4 ×8 doses) (61). The CR rate for MT-R induction was 52%. After a median follow-up of 79 months, the 2-year PFS and OS were 45% and 58%, respectively. For patients receiving EA consolidation, the 2-year PFS and OS were 78% and 93%, respectively. All patients developed grade 4 neutropenia and thrombocytopenia. There was no grade 3 or 4 neurotoxicity and no treatment-related deaths during consolidation therapy.

In the CALGB 50202 study, 44 patients with newly diagnosed PCNSL were treated with induction therapy with the MT-R regimen (methotrexate 8 g/m^2^, iv, day 1, ×8 cycles; rituximab 375 mg/m^2^, iv, day 3, cycles 1–6; TMZ 150 mg/m^2^, po, days 7–11, odd cycles), and patients who achieved CR received EA consolidation (etoposide 40 mg/kg, iv, days 1–4; cytarabine 2 g/m^2^, iv, over 2 h every 12 h ×8 doses) (62). The rate of CR under the MT-R regimen was 66%. The overall 2-year PFS was 57%, with a median follow-up time of 4.9 years. The 2-year time to progression was 59%, and for patients who completed consolidation, it was 77%. For patients aged 60 years as well as younger patients, the most significant clinical prognostic variable was treatment delay. High BCL6 expression was correlated with shorter survival.

### Radiotherapy

#### Whole-Brain Radiotherapy (WBRT)

In a previously mentioned phase II study, the median follow-up for living eligible patients was 3.6 years, and the 2-year OS and PFS were 80.8% and 63.6%, respectively (55). In phase II, the ORR was 85.7%. Among the 53 patients, 66% experienced grade 3 and 4 toxicities before WBRT, and 45% had grade 3 and 4 toxicities due to post-WBRT chemotherapy. The cognitive function and quality of life of these patients were either significantly improved or stabilized after WBRT.

#### Hippocampal-Avoidance WBRT (HA-WBRT)

The hippocampus plays a crucial role in episodic memory processing (63). Gondi et al. demonstrated that adult brain cancer patients could experience a significant decline in delayed recall after receiving a dose of over 7.3 Gy to 40% of the hippocampus, suggesting the harmful effects of radiation on the hippocampus (64). Hippocampal-avoidance WBRT (HA-WBRT) is a new option to prevent hippocampal damage. Recently, many studies have shown the clinical efficacy of HA-WBRT on brain metastases (65–69). Although there is a lack of clinical data about HA-WBRT for PCNSL, it is still a promising choice for PCNSL.

### Surgery

The role of surgery in PCNSL is generally restricted to stereotactic biopsy due to widespread and diffusely infiltrative tumor growth. Surgical resection increases the risk of permanent neurologic deficits in a disease that often involves deep structures and is highly chemo-sensitive. No survival benefit from subtotal or gross total resection has been observed in retrospective studies (70), but recently, this view has been challenged by several more recent studies (71–73). Gamma knife stereotactic radiosurgery in combination with MTX provides a promising strategy for the treatment of PCNSL (73). Surgery plus adjuvant radiotherapy seems to be a potential choice for PCNSL (74). However, there is insufficient evidence to recommend an aggressive surgical approach, including resection, for PCNSL.

### Autologous Stem-Cell Transplantation (ASCT)

A retrospective study of 48 central nervous system lymphoma (CNSL) patients, who have received high-dose chemotherapy and ASCT using a TBC (thiotepa, busulfan, cyclophosphamide) conditioning regimen, showed that the 2-year PFS and OS were 80.5% and 80.1%, respectively (75). The 2-year PFS and OS for patients with PCNSL in CR1 were 95.2% and 95.2%, respectively. Treatment-related mortality (TRM) was 8.3% and was mainly treatment-related overwhelming infection in the first 100 days post-transplantation. These data support the premise of consolidative ASCT for patients with PCNSL or SCNSL.

In a report on 64 patients with PCNSL, 38 patients were initiated on the transplantation-eligible protocol, of whom 30 underwent successful ASCT, and 26 were deemed transplantation ineligible. Out of those 26 patients, only 7 completed the transplantation-ineligible HD-MTX-based regimens (76). The transplantation protocol was HD-MTX/cytarabine-based induction followed by ASCT using a thiotepa and busulfan (TBu) conditioning regimen. For the transplantation-eligible and transplantation-ineligible cohorts, the projected 3-year OS rates were 83.8% and 14.3%, and the PFS rates were 78.1% and 0%, respectively. Among the 30 patients who underwent TBu/ASCT, the 3-year OS and PFS were 92.7% and 88.9%, respectively, without TRM or obvious neurotoxicity. These practical results highlight the efficacy and tolerability of TBu/ASCT consolidation for PCNSL in young patients who are fit enough for an intensive treatment program, along with the significant need for improved therapies for older or less fit patients with PCNSL.

An open-label, multicentric, non-randomized, single-arm phase II trial in 12 German centers testing the safety and effectiveness of an age-adapted induction treatment followed by HDT-ASCT in the elderly and fit PCNSL patients is undergoing a clinical trial (German clinical trials registry DRKS00011932) (77). Fifty-one immunocompetent PCNSL patients will be enrolled in this study and will be given age-adapted induction treatment (MAR) followed by ASCT using a conditioning regimen (rituximab 375 mg/m^2^, day −8; busulfan 3.2 mg/kg/day, days −7 to −6; thiotepa 5 mg/kg/day, days −5 to −4). The primary endpoint was 1-year PFS and the secondary endpoints were the rate of CR on day +30 post-HDT-ASCT, PFS, OS, QoL, and non-relapse mortality (NRM).

In a randomized phase II PRECIS study (NCT00863460) in 23 French centers, 140 immunocompetent PCNSL patients were randomly assigned to receive consolidative WBRT or ASCT after induction chemotherapy consisting of two cycles of R-MBVP followed by two cycles of R-Ara C (78). The conditioning regimen consisted of thiotepa, busulfan, and cyclophosphamide. WBRT delivered 40 Gy. The 2-year PFS rates were 63% and 87% in the WBRT and ASCT arms, respectively. Toxicity deaths were recorded in 1 and 5 patients after WBRT and ASCT, respectively. Cognitive impairment was observed after WBRT, whereas cognitive functions were preserved or improved after ASCT.

A retrospective study enrolled 46 PCNSL patients in CR1 who underwent transplantation using a TBC-based conditioning regimen. Fifty-nine percent of the induction regimens were HD-MTX plus TMZ and rituximab (79). No patients received WBRT. Forty patients received cytarabine before undergoing ASCT as either induction intensification, early consolidation therapy, or mobilization. With a median follow-up of 2.7 years after ASCT, the 2-year OS and PFS were 95% and 92%, respectively. The most common toxicities were severe mucositis (35%) and bacterial infections within 100 days after transplantation (35%). The estimated 2-year non-recurrence mortality rate was 2.9%.

Hyung et al. have compared the CR or PR of PCNSL patients after induction chemotherapy who received a TBC conditioning regimen with those who received a BuCyE conditioning regimen before ASCT (80). The TBC cohort received thiotepa (250 mg/m^2^) on days −9 to −7, busulfan (3.2 mg/kg) on days −6 to −4, cyclophosphamide (60 mg/kg) on days −3 to −2, and stem cell infusion on day 0. The BuCyE cohort was administered busulfan (3.2 mg/kg) on days −7 to −5, etoposide (200 mg/m^2^) on days −5 to −4, cyclophosphamide (50 mg/kg) on days −3 to −2, and stem cell infusion on day 0. The 2-year OS rates were 88.1% and 64% in the TBC and BuCyE arms, respectively. The 2-year PFS rates were 84.7% and 40% in the TBC and BuCyE arms, respectively. Adverse effects of grade 3 or 4 toxicities, including mucositis, diarrhea, and nausea, were significantly more frequent in the TBC group than in the BuCyE group. There was no case of TRM. A hemorrhagic complication was reported in one patient in the TBC group. Previous clinical studies have demonstrated that the BEAM regimen is not recommended as a conditioning regimen for PCNSL.

A seemingly contradictory outcome came out of another study: 38 consecutive older immunocompetent PCNSL patients were treated with an R-MPV/Ara-C regimen followed by consolidative WBRT or ASCT and patients aged <60 years and >60 years had similar ORRs of 100% and 85%, 4-year PFS rates of 81% and 82%, and 4-year OS rates of 80% and 77%, respectively (81). This study suggests that older patients with PCNSL might be effectively treated with sequential and response-adapted MTX dosing without the need for WBRT or ASCT.

In contrast to BEAM or BUCYE, thiotepa has an excellent penetration ability through the central nervous system, with CSF levels greater than 80%, and has been adopted in combination with busulfan, cyclophosphamide, and carmustine in different conditioning regimens (82). Recently, an observational cohort study reported by the Center for International Blood and Marrow Transplant Research (CIBMTR) gave 603 adult PCNSL patients ASCT as either initial or subsequent consolidation therapy (83). Patients were given any one of the following three conditioning regimens: TBC, thiotepa/carmustine (TT-BCNU), and BEAM. In comparison to the BEAM group, the TBC and TT-BCNU groups showed significantly higher 3-year adjusted PFS rates. In a multivariable regression analysis, the TT-BCNU group exhibited a significantly higher relapse risk, lower risk of NRM, and similar risk of all-cause mortality more than 6 months after ASCT compared with the TBC group. Age ≥ 60 years, KPS< 90, and HCT-CI ≥ 3 were correlated with lower rates of survival among all three groups. Subgroup analyses showed that patients aged ≥ 60 years exhibited higher NRM with TBC conditioning.

Together, the evidence recommends consolidation therapy with ASCT based on a thiotepa-containing conditioning regimen for those who achieved CR after induction therapy. There is a lack of clinical data about allo-SCT for PCNSL.

### CAR T Therapy

Chimeric antigen receptor (CAR) T-cell therapies utilize the patients’ own T cells that have been genetically engineered to bind to a specific antigen on target cancer cells, such as CD19 protein, which is expressed on most B-cell leukemias and lymphomas (84, 85). CAR T-cell therapy has shown encouraging results in R/R DLBCL, with CR rates higher than 50% (86).

A preclinical study investigated the effects of anti-CD19 CAR T cells on PCNSL in animal models (87). Intravenous injection of anti-CD19 CAR T cells resulted in poor infiltration into the tumor and could not efficiently control tumor growth. However, intracerebral injection caused anti-CD19 CAR T cells to invade deeply into the solid tumor, suppressed tumor growth, and led to regression of PCNSL, which were associated with long-term survival. Intracerebral anti-CD19 CAR T cells entered the blood circulation and infiltrated distant, non-draining lymph nodes more efficiently than mock CAR T cells. After CR of the tumor, anti-CD19 CAR T cells remained detectable intracranially and intravascularly for nearly 159 days. Collectively, these results suggest the great potential of anti-CD19 CAR T cells for the treatment of PCNSL.

However, the information on practical clinical treatment with CAR T cells for PCNSL is limited. One paper reported that a 67-year-old male PCNSL patient was effectively treated with 4SCAR19 and 4SCAR70 (T cells were transduced with a safety-engineered lentiviral vector encoding a fourth-generation CAR containing anti-CD19 or anti-CD70 scFv fused with multiple intracellular signaling domains) (CD28-CD27-CD3z-2A-iCasp9) (88). This patient received lymphodepletion chemotherapy containing fludarabine (30 mg/m^2^/day) and cyclophosphamide (300 mg/m^2^/day) on days −4 to −2, followed by the infusion of 1 × 10^8^ CAR19 T cells and 8.2 × 10^7^ CAR70 T cells on day 0. After 17 months, MRI suggested a durable CR in this patient. Both CAR19 and CAR70 T cells were detectable more than 10 months after CAR T-cell infusion. This case shows the promising efficacy of CAR T cell infusion for the treatment of PCNSL. Several clinical trials (NCT04443829, NCT04608487, NCT04134117, and NCT03484702) testing the usage of CAR T cells for the treatment of PCNSL are underway (**Table 1**).

## Conclusions and Future Challenges

**Figure 1:**
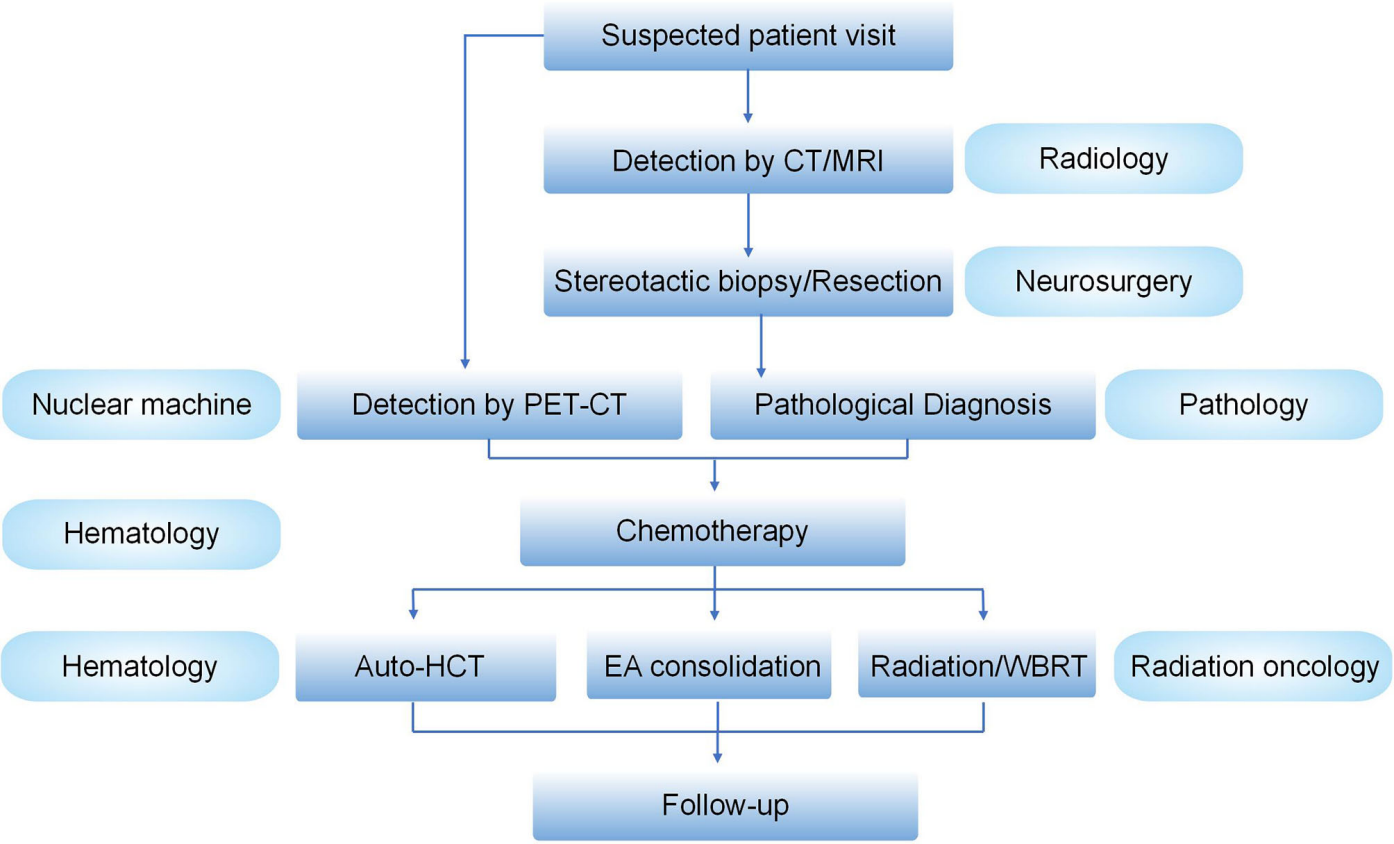
Management schedule for an experienced MDT for PCNSLs. Suspected PCNSL patients visit the clinics, followed by the detection of brain by CT/MRI. If there is a preferred mass in the brain, the patients will receive stereotactic biopsy or resection by neurosurgery. The pathological diagnosis will be made by the Department of Pathology. PET/CT will be mainly used to differentiate the PCNSL from secondary CNSL. After that, the condition of the confirmed patients will be further evaluated and the precise treatment will be discussed by hematologists and irradiation oncologists in an MDT meeting. After the treatment, patient follow-up will be made.

PCNSL is a rare extra-nodal lymphoma. The precise diagnosis of PCNSL needs a strong advancements in multidisciplinary cooperation, at least including the neurosurgery, radiology, pathology, and hematology departments. The main treatment options for PCNSL are summarized in **Table 2**. Although HD-MTX-based chemotherapy in combination with BTKi or lenalidomide, followed by ASCT or radiotherapy, significantly improved the survival rate of PCNSL patients, a series of questions remain to be resolved. First, for patients administered with MTX-based chemotherapy, knowledge of the best number of cycles of chemotherapeutic regimens in induction therapy is of profound importance. How many cycles? What is the best drug combination? Second, do the treatment strategies vary between different subtypes, e.g., DLBCL- *vs*. NK-T-PCNSL? Third, the best choice for the consolidation therapy is still uncertain but selecting either conventional chemotherapy, ASCT, WBRT, or CAR T treatment is crucial (91, 92). Fourth, there is still no gold-standard comprehensive evaluation system for the treatment effects. Recent evaluation methods only contain two separate systems: imaging evaluation for the mass size and cognitive function evaluation (93, 94). Notably, the management of PCNSL has required an experienced multidisciplinary team (MDT) involving radiology, neurosurgery, hematology, pathology, and radiation oncology (**Figure 1**).

**Table 2:** Recent treatment options for PCNSL.

	Regimens	Agents	Number of patients	Outcomes	Adverse effects	References
Induction	MTX/ cytarabine combinations	Methotrexate, cytarabine or MATRix regimen (Methotrexate, cytarabine, thiotepa and rituximab)	118	1) CR rate: 49%	Infection (6.8%)	(89)
				2) The 2-year PFS rate: 11%		
				3) The OS rate: 24%		
				4) TRM: 6·8%		
	MT regimen	Methotrexate, temozolomide	30	1) ORR: 69.0%	1) No toxic death	(57)
				2) CR rate: 27.6%	2) Grade 3–4 hematological toxicity: 36.7%	
				3) The 2‐year PFS rate: 46.5%	3) Grade 3–4 non‐hematological toxicities: nausea/vomiting (6.7%), pneumonia (6.7%),	
				4) The 5‐year PFS rate: 29.1%		
				5) The 2‐year OS rate: 82.3%		
				6) The 5‐year OS rate: 82.3%	hepatotoxicity (3.3%), cardiotoxicity (3.3%)	
	RMT regimen	Rituximab, methotrexate, temozolomide	32	1) ORR: 93.7%	1) No toxic death	(57)
				2) CR rate: 53.2%	2) Grade 3–4 hematological toxicity: 34.4%	
				3) The 2‐year PFS rate: 81.3%	3) Grade 3–4 non‐hematological toxicities: pneumonia (12.5%), hepatotoxicity (3.1%)	
				4) The 5‐year PFS rate: 53.3%		
				5) The 2‐year OS rate: 65.7%		
				6) The 5‐year OS rate: 50.0%		
	R-MPV	Induction: Rituximab, methotrexate, procarbazine, vincristine	10	1) The median PFS: 57 m	Grade ≥3 toxicities:	(58)
		Maintenance: TMZ		2) The 5-year PFS rate: 33%	thrombocytopenia	
				3) The median OS: 63 m		
				4) The 5-year OS rate: 57%		
Consolidation	ASCT+TBC conditioning regimen	Conditioning regimen: thiotepa, busulfan, cyclophosphamide	46	1) The 2-year OS rate: 95%	Severe mucositis (35%) and bacterial infections	(79)
				2) The 2-year PFS rate: 92%		
			48	1) The 2-year PFS rate: 80.5%	Treatment-related infection	(75)
				2) The 2-year OS rate: 80.1%		
				3) TRM: 8.3%		
			28	1) The 2-year OS rate: 88.1%	Grade ≥3 toxicities: mucositis (96.4%), diarrhea (92.9%), nausea (85.7%)	(80)
				2) The 2-year PFS rate: 84.7%		
				3) TRM: 0%		
	ASCT+TBu conditioning regimen	Conditioning regimen: thiotepa and busulfan	30	1) The 3-year OS rate: 92.7%	Grade 3–4 toxicities: febrile neutropenia (70.0%), bacteremia (30.0%), mucositis (13.3%)	(76)
				2) The 3-year PFS rate: 88.9%		
				3) TRM: 0%		
	ASCT+BuCyE conditioning regimen	Conditioning regimen: busulfan, etoposide, cyclophosphamide	25	1) The 2-year OS rate: 64%	1) Usage of prophylactic antimicrobial agent: 64%	(80)
				2) The 2-year PFS rate: 40%	2) Grade ≥3 febrile neutropenia: 80%	
				3) TRM: 0%		
	WBRT + cytarabine	Induction: HD-MTX Consolidation: WBRT and cytarabine	41	1) ORR: 85.4%	1) Grade ≥3 hematologic toxicities: 4.8%	(90)
				2) CR rate: 60.9%	2) Grade ≥ 3 non-hematologic toxicities: 17.1%	
				3) The median PFS: 35.2 m		
				4) The median OS: 46.5 m		
				5) TRM rate: 2.4%		

Taken together, although there are a variety of choices for the treatment of PCNSL with the advent of BTKi and CAR T cells, many questions regarding the optimization of treatment strategies still need to be addressed.

## Author Contributions

LS drafted the manuscript. HW and SP completed the figures. MJ and LS did the language editing. CX, HW, FC, SL, DY, KL, and YW took part in the discussion and revision of the manuscript. All authors contributed to the article and approved the submitted version.

## Funding

This work was supported by the Natural Foundation of Hubei Province (China) (No.2017CFB631), The Innovative Foundation of Zhongnan Hospital of Wuhan University (China) (No.znpy2018025) and Science, Technology and Innovation Seed Fund of Oncology of Zhongnan Hospital of Wuhan University (No.ZNYY202101).

## Conflict of Interest

The authors declare that the research was conducted in the absence of any commercial or financial relationships that could be construed as a potential conflict of interest.

## Publisher’s Note

All claims expressed in this article are solely those of the authors and do not necessarily represent those of their affiliated organizations, or those of the publisher, the editors and the reviewers. Any product that may be evaluated in this article, or claim that may be made by its manufacturer, is not guaranteed or endorsed by the publisher.

## Abbreviations

PCNSL, Primary CNS lymphoma; CAR T, Chimeric antigen receptor T; BTKi, Bruton’s tyrosine kinase inhibitor; ASCT, Autologous stem-cell transplantation; DWI, Diffusion-weighted imaging; ADC, Apparent diffusion coefficient; rCBV, relative cerebral blood volume; DSC-MRI, Dynamic Susceptibility Contrast Perfusion MR Imaging; DCE-MRI, Dynamic Contrast Enhanced Perfusion MR Imaging; TMZ, temozolomide; DLT, dose-limiting toxicity; DLBCL, diffuse large B-cell lymphoma; NHL, non-Hodgkin lymphomas; BBB, blood–brain barrier; HIV, immunodeficiency virus; IRF4, interferon regulatory factor 4; GCB, germinal center B cell; non-GCB, non-germinal center B cell; CNAs, copy number alterations; PI3K, Phosphatidylinositol 3-kinase; mTOR, mammalian target of rapamycin; RT, radiotherapy; TMB, tumor mutational burden; CR, complete remission; TIL, tumor-infiltrating lymphocyte; MTD, maximum tolerated dose; PTLpr, imary testicular lymphomas; PFS, progression-free survival; ORR, objective response rate; OS, overall survival; TAMs, tumor-associated macrophages; CARD11, Caspase Recruitment Domain Family Member 11.
